# Fertility intentions among young people in the era of China’s three–child policy: a national survey of university students

**DOI:** 10.1186/s12884-022-04873-y

**Published:** 2022-08-12

**Authors:** Chenyun Zhang, Lingling Wei, Yinyan Zhu, Li Teng, Wenchang Zhang, Jia Xu, Mengqi Qin, Na Jiang, Haridah Alias, Li Ping Wong

**Affiliations:** 1 Department of Health Law and Policy, School of Public Health, Fujian Medical University, Fuzhou, 350122 Fujian Province China; 2 Fujian Provincial Key Laboratory of Environment and Health, Fujian Medical University, Fuzhou, 350122 Fujian Province China; 3 Psychological Health Education and Counseling Center, Xiamen City University, Xiamen, 361000 Fujian Province China; 4 School of Management, North Sichuan Medical College, Nanchong, 637000 Sichuan Province China; 5 School of Public Health, Dalian Medical University, Dalian, 116044 Liaoning Province China; 6 The First Hospital of Lanzhou University, Lanzhou, 730000 Gansu Province China; 7 Centre for Epidemiology and Evidence-Based Practice, Department of Social and Preventive Medicine, Faculty of Medicine, Universiti Malaya, 50603 Kuala Lumpur, Malaysia

**Keywords:** Three–child policy, Fertility intentions, Young adult, Childbearing, Parenthood

## Abstract

**Background:**

This study aimed to assess the fertility intentions of young people after the announcement of the three–child policy in China and to determine whether knowledge about reproductive, maternal, newborn, and child health (RMNCH) services or support, childbearing- and childbirth-related anxiety, and parenthood–related anxiety influence fertility intentions.

**Methods:**

A cross-sectional Internet-based survey was conducted on a nationwide sample of young people aged 18 to 28 years old in education institutions. Factors associated with fertility intentions were analysed using partial least squares structural equation modelling (PLS-SEM).

**Results:**

Only 4.2% of males and 1.7% of females intended to have three children or more. On the whole, the majority (40.3%) reported the intention to have two children. The mean and standard deviation (SD) for the total knowledge RMNCH support and/or services knowledge score was 9.5 (SD ± 8.9), out of a possible score of 39. The median and interquartile range (IQR) of childbearing– and childbirth-related anxiety score was 8.0 (IQR = 6.0–9.0), out of a possible score of 10. The median and IQR of parenthood–related anxiety score among the males was 6.0 (IQR = 4.0–9.0) and for females was 7.0 (IQR = 5.0–9.0). Results from PLS-SEM revealed that a higher level of knowledge of RMNCH support and/or services is significantly associated with higher fertility intentions. Both childbearing- and childbirth-related anxiety and parenthood–related anxiety were inversely associated with fertility intentions.

**Conclusion:**

Raising awareness about RMNCH supportive measures and easing birth- and parenting anxiety are imperative to enhance birth rates. Future policies should pay more attention to these determinants to achieve their intended goal of boosting population growth.

**Supplementary Information:**

The online version contains supplementary material available at 10.1186/s12884-022-04873-y.

## Background

In China, the relaxation of the one-child policy in 2013, and its termination in 2015 generated only a small and temporary increase in the fertility rate. On 31 May 2021, the authorities in China announced that couples can have up to three children, ending the two-child policy that has not been successfully raised the country’s continuous declining birthrates. The announcement comes after the release of the results of the Seventh National Population Census [1], which showed that the number of births in mainland China in 2020 was only 12 million, the lowest number of births in the country since 1962. Alarmingly, the census also revealed China’s total fertility rate of women of childbearing age declined from 1.6 in 2017 to only 1.3, which is lower than that of many developed countries, such as Japan at 1.4 [2], the European Union at 1.5 and the United States at 1.7 [2, 3]. The declining fertility rate may result in a shrinking working-age population and a growing retired population that may not only hamper China’s economic growth but also strains social services. As the world’s most populous country, China has remained the main player in driving the global economy. Hence, from the international perspective, China’s declining population is also of global significance.

In October 2015, when China’s one-child policy was replaced by a universal two-child policy, the country experienced a short–term boost in birth rates for two years [4]. When the two-child policy was introduced, studies revealed that the majority of women desired one or two children, while in large cities nearly two–thirds of women stated a preference for only one child [5]. Recently published findings from the 2017 National Fertility Survey in China reported the intended number of children is 1.76 on average [6]. Since then studies continuously found declining fertility intentions among the Chinese public. A recently published study on the population of Dalian City reported the average intended number of children of 1.73 [7]. A broad range of fear and anxiety was reported, among which the most prominent were high child-rearing costs, particularly in child education, the effect on lifestyle, and interference in the mother’s career development [5]. Hence, along with the current announcement of the new three-child policy, the government is reported to also be working on improving supportive measures that may facilitate couples to have more children and encourage births, covering various aspects of childbearing, childcare, parenting, education, taxation, housing, and women’s rights in employment [8].

In recent years, many supporting measures are being designed to improve the reproductive, maternal, newborn, and child health (RMNCH) of the communities in China [9]. Young people have a positive and important impact on driving the country’s population growth. Promoting utilization of RMNCH services and/or support in young people is a milestone in ensuring sustainable reproductive health care and towards achieving targeted family planning and population control. It has been previously reported that knowledge and awareness are among the main barriers affecting demand for RMNCH services [10]. Therefore, having adequate knowledge of the local RMNCH services will help improve rational fertility decision–making and reduce decisional conflict among couples. Young people’s knowledge and awareness of the RMNCH, as well as their support and/or services, are largely unknown in China hence require urgent investigation.

There has not been much empirical research in China that examines the perception of the young generation about their fertility intentions after the recent announcement of the three–child policy. With the current new reform, whether the three-child policy is well supported by young people is worthwhile exploring. It is important to gauge the fertility intentions of young people as they are the contributors to future population growth. Henceforth, this study aimed to assess the fertility intentions of young Chinese men and women in response to the newly announced three-child policy. Secondly, factors (childbearing- and childbirth-related anxiety, parenthood–related anxiety, and knowledge of the RMNCH support and/or services) influencing fertility intentions were also investigated. We hypothesized that people with a lack of knowledge about RMNCH support and/or services, and a high level of childbearing- and parenthood anxiety would likely have lower fertility intentions.

## Methodology

### Study participants and survey design

A nationwide cross-sectional study was conducted from 30th August to 25th September 2021, through the dissemination of an online survey to educational institutions in all regions in mainland China. The inclusion criteria were young unmarried people or youth, and Chinese citizens. The United Nations defines youth as 15–24 years of age. As the legal participation age in a survey is 18 years or above, the minimum age set for participation is 18 years old. In China, the majority of the young people aged 18 to 24 are still attending colleges or universities. The literacy rate of young people aged 15–24 years in China was reported as 99.78% [11]. Hence, the online survey was promoted through social media and networking across all higher educational institutions throughout all the regions in China. As China’s main economic regions are divided into north, northeast, east, south-central, southwest and northwest regions, samples were collected over the six regions. The sample size was calculated for each region using the formula: *n* = Z^2^ P(1–P)/d^2^ where Z = 1.96 for a confidence level of (α) of 95%, *P* = % of population probability, assumed to be 50%, d = margin of error of 0.05 Using a margin of error of 0.05 (5%), with a 95% CI and 50% response distribution, the calculated sample size was 384 for each region. The sample size was multiplied by the predicted design effect of two to account for the use of convenience sampling and an online survey. Hence, the optimal sample size for each region was set to 768 (384 × 2).

### Instruments

The questionnaire (Supplementary File 1) consisted of four parts: 1) demographic background; 2) fertility intentions; 3) childbearing– and childbirth-related anxiety; 4) parenthood-related anxiety; and 5) knowledge about RMNCH support and services.

#### Demographics background

Personal details collected include age, gender, current study grades, paternal and maternal highest educational level, monthly household income and current residing region.

#### Fertility intentions

The participants were questioned about the desired number of children they would want if they are starting a family in the future. Option answers were ‘*I do not intend to start a family*’, ‘*I do not intend to have any children if I start a family*’, ‘*one child*’, ‘*two children*’ ‘*three children*’ and ‘*more than three children*’.

#### Childbearing– and childbirth-related anxiety

Only female participants answered the assessment of childbearing and childbirth anxiety. They were asked to rate their level of childbearing– and childbirth-related anxiety on a scale of 0 to 10, with a higher scale indicating greater anxiety. Subsequently, they were queried on which aspects of childbearing– and childbirth-related anxiety they perceived as most fearful. The option answers were ‘*childbearing/pregnancy process*’, ‘*child delivery process*’, ‘*personal health risk*’, ‘*infant’s health* (including foetal anomalies or birth defects)’ and ‘*cost/associated expenses*’.

#### Parenthood–related anxiety

Parenthood–related anxiety was assessed in both male and female participants. They were asked to rate their level of anxiety on a scale of 0 to 10, with a higher scale indicating greater anxiety. Likewise, the participants were assessed regarding which aspects of parenthood they perceived as most fearful. The option answers were ‘*balancing work and childcare*’, ‘*loss of freedom*’, ‘*loss of self–identity*’, ‘*interfere relationship with spouse*’ and ‘*cost/associated expenses*’.

#### Knowledge about reproductive, maternal, newborn, and child health (RMNCH) support and/or services

A self–developed questionnaire was used to assess participants’ level of knowledge and awareness of the RMNCH support and/or services in their local area. The question items were developed based on the recent guidelines on optimizing fertility policies and promoting long–term balanced population development by the Communist Party of China (CPC) Central Committee and State Council [9]. The 13–item questions were content validated by content experts who are professionals who have research experience or work in the field related to child and infant care and population development policies. Participants were given ‘not aware’, ‘aware but don’t know much’, ‘knowledgeable’ or ‘very knowledgeable’ response options to the 13 items. The score of each option answer was assigned as 0, 1, 2, and 3, respectively. The maximum total score ranged from 0–39, with a higher score indicating better knowledge of RMNCH support and services.

### Statistical analysis

Results are presented in tables, charts and graphs, as appropriate. Categorical variables were described as frequencies and percentages, and numerical variables as mean or median. Descriptive statistics and bivariate were performed using SPSS version 12.0. Associations with *p* < 0.05 were considered statistically significant. Partial least squares structural equation modelling (PLS-SEM) was used to quantify the contributing factors (socio-demographics, childbearing– and childbirth-related anxiety, parenthood–related anxiety, and knowledge of RMNCH support and/or services) of fertility intentions. Fertility intentions (dependent variable) were coded as 1– do not intend to start a family or do not intend to have any children, 2–one child, 3–two children, and 4–more than three children. Bootstrapped models were implemented to examine whether the associations evident in the calculated specifications were significant. This technique assesses the statistical significance and the relevance of the path coefficients, and the error of the estimated path coefficients [12]. The bootstrapped significance calculation was conducted using SmartPLS software version 3.2.8 (SmartPLS GmbH) [13]. Results of the measurement model indicated that all the 13 indicators for the knowledge of RMNCH support and/or services had adequate construct reliability (CR = 0.970) and convergent validity (average variance extracted, AVE = 0.710). The variance inflation factors (VIFs) for all indicators were below 2.5, indicating that all indicators belonging to the construct were adequately independent. Discriminant validity assessment through heterotrait-monotrait (HTMT) ratio of correlations method also indicated that all HTMT values were lower than the most restrictive threshold (0.85), thus indicating adequate discriminant validity [14].

## Results

**Fig. 1:**
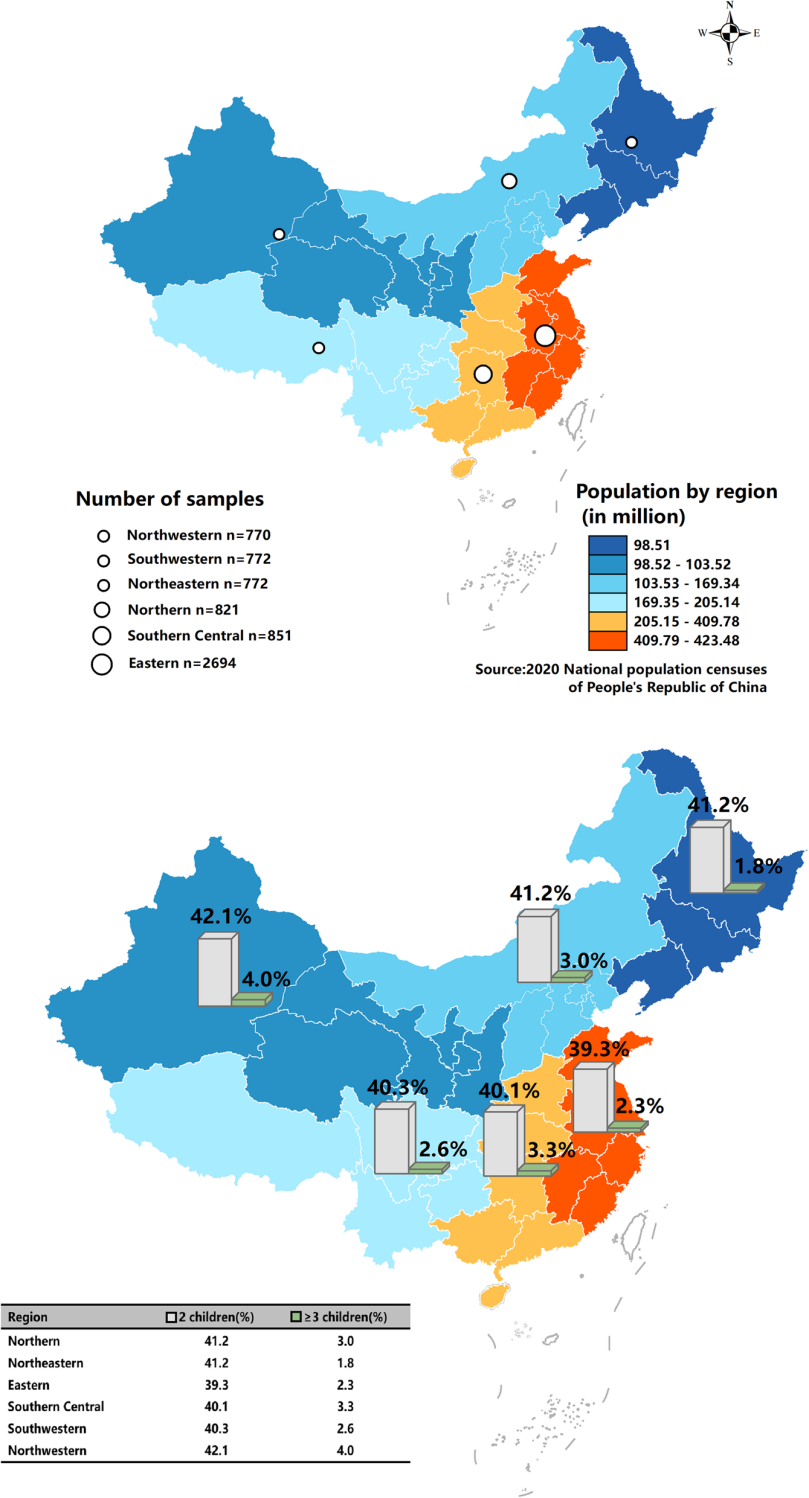
Distribution of fertility intentions by region

In total, 6680 responses from six regions in China were received. The total sample size obtained by region ranges from 770 to 2694. The obtained responses from regions were higher than the calculated optimal sample size. The highest response was obtained from the East region (40.3%) and the lowest was from the Northeast (11.6%) and Southwest (11.6%) regions (Fig. 1). The demographics of study participants are presented in Table 1. The majority of study participants were in their second (30.9%) and third (24.3%) grades and between the age of 18 and 20 years (48.7%). The average age of all study participants was 20.3 ± 1.9. As the survey link was sent out to all higher educational institutions, a small proportion of post-graduate students responded to the survey. In total, 10.3% of the study participants were of the age 24–28 years old. Nearly half (47.7%) was from a family with a monthly household income of CNY ¥ 4000–9999.

**Table 1:** Demographic characteristics of study participants (*N* = 6680^a^)

	Overall (*N* = 6680)	Female (*n* = 4025)	Male (*n* = 2655)
**Socio–demographic characteristics**			
Age group (years)			
18–20	3252 (48.7)	1942 (48.2)	1310 (49.3)
21–23	2737 (41.0)	1639 (40.7)	1098 (41.4)
24–28	691 (10.3)	444 (11.0)	247 (9.3)
Study grade			
Grade 1	733 (11.0)	425 (10.6)	308 (11.6)
Grade 2	2063 (30.9)	1167 (29.0)	896 (33.7)
Grade 3	1625 (24.3)	946 (23.5)	679 (25.6)
Grade 4/5	1300 (19.5)	809 (20.1)	491 (18.5)
Postgraduate	959 (14.4)	678 (16.8)	281 (10.6)
Type of institution			
Undergraduate college	4943 (74.0)	3068 (76.2)	1875 (70.6)
Vocational school	1737 (26.0)	957 (23.8)	780 (29.4)
Maternal highest education level			
Primary school	2023 (30.3)	1253 (31.1)	770 (29.0)
Junior middle school	1990 (29.8)	1258 (31.3)	732 (27.6)
Secondary school/ high school	1172 (17.5)	683 (17.0)	489 (18.4)
College/ university	1495 (22.4)	831 (20.6)	664 (25.0)
Paternal highest education			
Primary school	1175 (17.6)	723 (18.0)	452 (17.0)
Junior middle school	2375 (35.6)	1466 (36.4)	909 (34.2)
Secondary school/ high school	1582 (23.7)	948 (23.6)	634 (23.9)
College/ university	1548 (23.2)	888 (22.1)	660 (24.9)
Monthly household income (CNY ¥)			
< 4000	1037 (15.5)	692 (17.2)	345 (13.0)
4000–9999	3189 (47.7)	2029 (50.4)	1160 (43.7)
10,000–14,999	1380 (20.7)	781 (19.4)	599 (22.6)
15,000 and above	1074 (16.1)	523 (13.0)	551 (20.8)

^a^Samples were from 31 provinces (excluding Hong Kong, Taiwan and Macau) in mainland China

### Fertility intentions

**Fig. 2:**
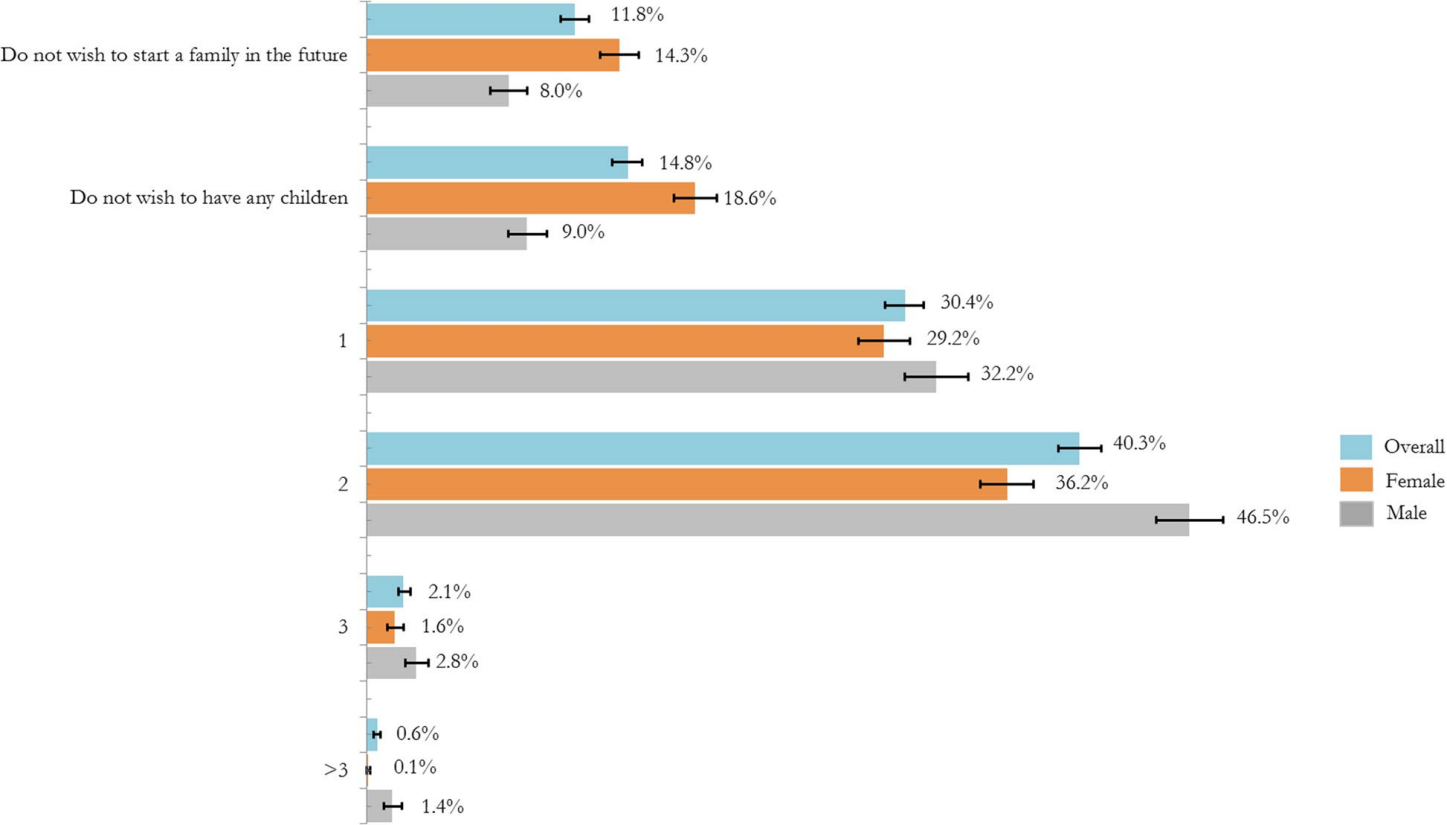
Fertility intentions by gender

Figure 1 shows the distribution of fertility intentions by region. There were no significant differences in fertility intentions by region. The highest proportion (4.0%) of participants from the northwestern region reported a wish to have three children or above. Findings on fertility intentions by gender in Fig. 2 showed that a small proportion of both male (2.8%) and female (1.6%) participants has an intention to have three children. On the whole, 40.3% desired to have two children, with a higher proportion being males (46.5%) compared to females (36.2%). A substantial proportion (26.6%) reported do not wish to start a family or not wanting any children in the future.

Knowledge about reproductive, maternal, newborn, and child health (RMNCH) support and/or services.

**Fig. 3:**
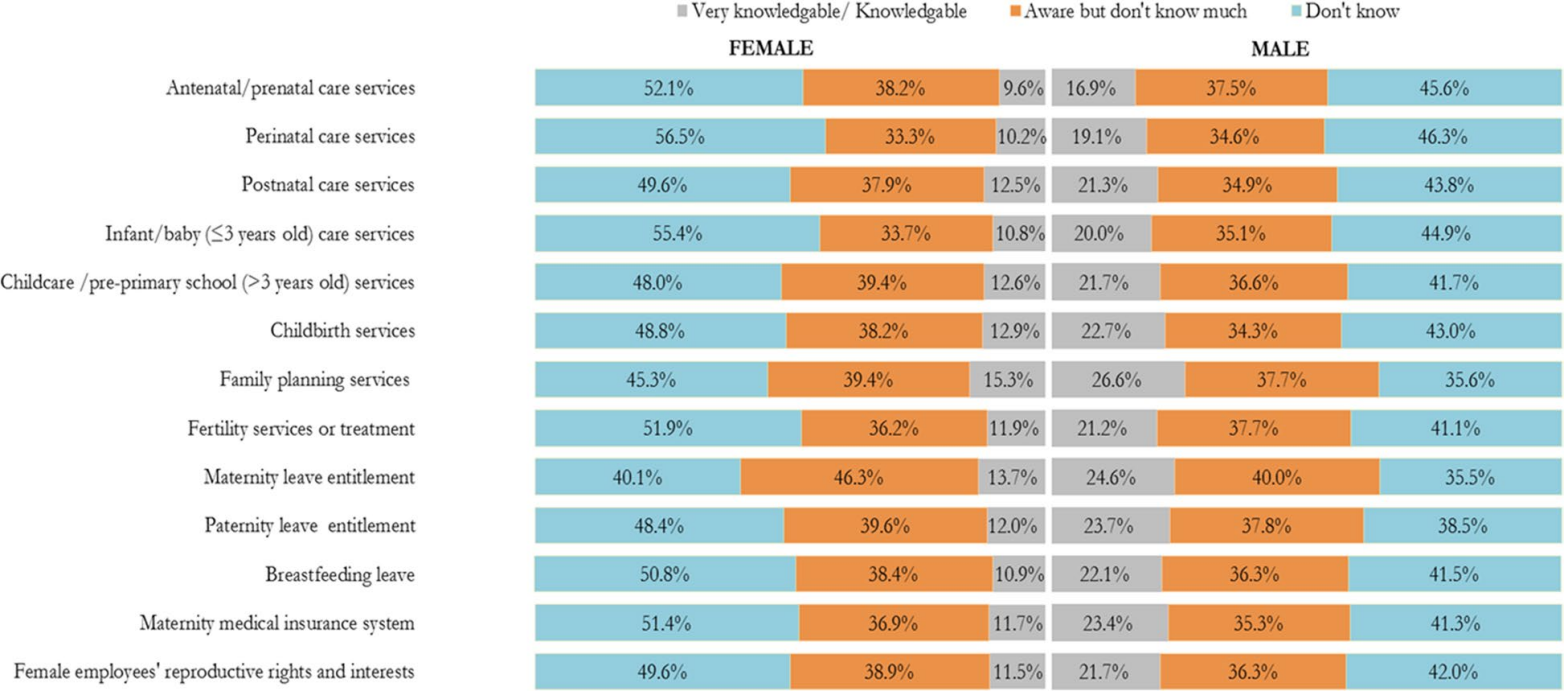
Proportion of level of knowledge about reproductive, maternal, newborn, and child health (RMNCH) support and/or services

Figure 3 shows the proportion of responses to the knowledge of RMNCH support and/or services items by gender. The majority of both male and female participants responded *Don’t know* and *Aware but don’t know much* in all the knowledge items. On the whole, a higher proportion of male participants reported *Very knowledgeable/knowledgeable* on all the item questions on knowledge of RMNCH support and/or services. A high proportion of female participants reported *completely not aware* of perinatal care services (56.5%), antenatal/prenatal care services (52.1%) and fertility services or treatment (51.9%) and breastfeeding leave for working mothers (50.8%).

The mean and standard deviation (SD) for the total knowledge RMNCH support and/or services score was 9.5 (SD ± 8.9), out of a possible score of 39. The median was 8 (interquartile range (IQR) 1.0–13.0). The knowledge scores of the study participants ranged from 0 to 39. The knowledge scores were categorized as a score of 8–39 or 0–7, based on the median split; as such, a total of 3537 (52.9%; 95% CI 51.7 to 54.2) were categorized as having a score of 8 to 39 and 3143 (47.1%; 95% CI 45.8 to 48.3) were categorized as having a score of 0–7. Higher monthly average household income, maternal and paternal educational levels were associated with a higher level of knowledge of RMNCH support and/or services. Males were found to have a higher level of knowledge of RMNCH support and/or services than females. A high level of knowledge of RMNCH support and/or services was found in participants from Northeastern and Northwestern regions (Supplementary File 2).

### Childbearing- and childbirth-related anxiety and parenthood–related anxiety

**Fig. 4:**
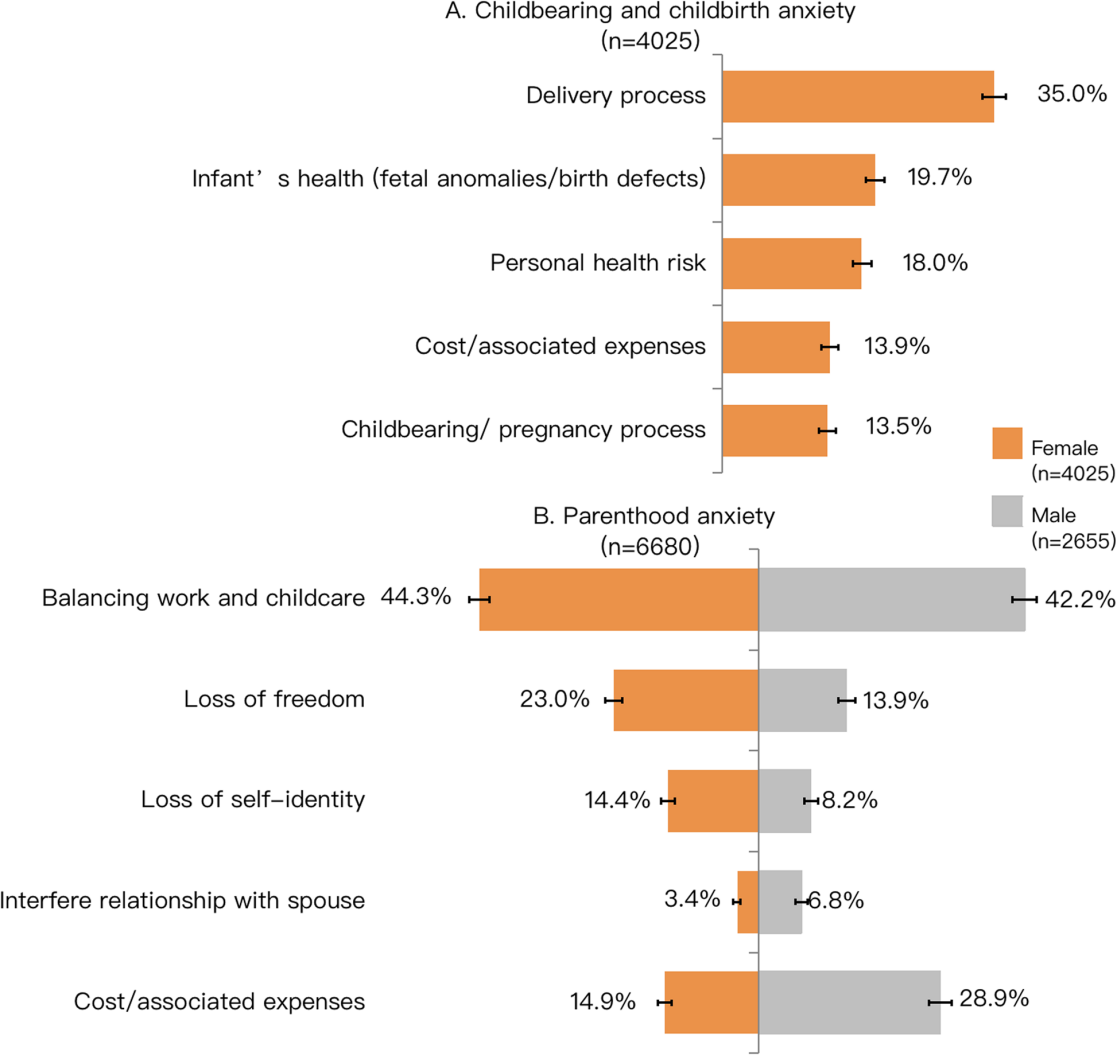
Distribution of childbearing- and childbirth-related anxiety and parenthood-related anxiety

The median and IQR of childbearing- and childbirth-related anxiety score was 8.0 (IQR = 6.0–9.0). A high proportion of female participants reported that the delivery process is the most fearful aspect of childbearing and childbirth (35.0%), followed by the infant’s health (19.7%) and personal health risk (18.0%) (Fig. 4). The median and interquartile range of parenthood–related anxiety score among the males was 6.0 (IQR = 4.0–9.0) and for females was 7.0 (IQR = 5.0–9.0). A high proportion of males (42.2%) and females (44.3%) regarded balancing childcare and work as the most fearful aspect of parenthood. Near 30% of males reported cost or associated expenses of parenthood as most fearful. A total of 23.0% of female participants reported loss of freedom and 14.9% viewed cost or associated expenses as the most fearful aspect of parenthood (Fig. 4).

### Factors associated with fertility intentions

**Fig. 5:**
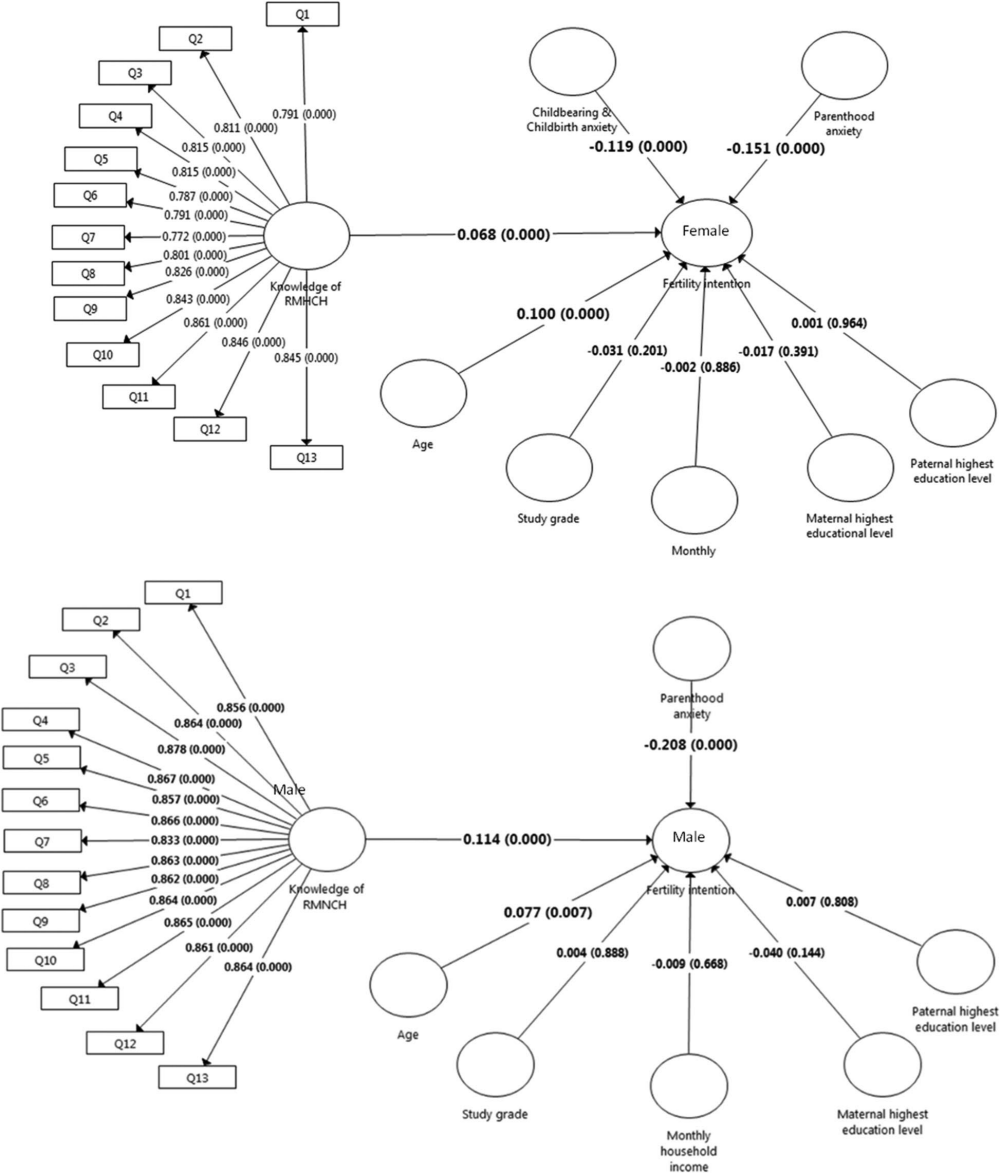
PLS Path Modelling for Factors Influencing fertility intention by gender

The PLS-SEM in Fig. 5 shows the hypothesized associations between the demographics, parenthood–related anxiety, childbearing- and childbirth-related anxiety and childbirth, knowledge of RMNCH support and/or services and fertility intentions for both male and female samples. The PLS-SEM path analysis revealed that older age is significantly associated with higher fertility intentions in both the males (*β* = 0.077; *p* < 0.01) and females (*β* = 0.100; *p* < 0.001). The higher *β* value in the females symbolizes the stronger effect of age towards fertility intentions compared to males. In the PLS-SEM path analysis for male samples, knowledge of RMNCH support and/or services is significantly associated with higher fertility intentions (*β* = 0.114; *p* < 0.01). Parenthood–related anxiety was inversely associated with fertility intentions (*β* = –0.208; *p* < 0.001). Parenthood–related anxiety had a stronger path coefficient than knowledge of RMNCH support and/or services. Similarly, the model for female samples shows that knowledge of RMNCH support and/or services is significantly associated with fertility intentions (*β* = 0.068; *p* < 0.001). As hypothesized, both childbearing- and childbirth-related anxiety (*β* = –0.119; *p* < 0.001), and parenthood–related anxiety (*β* = –0.151; *p* < 0.001) are inversely associated with fertility intentions. The results for adjusted R^2^ indicated that the models explained 6.5% and 7.5% of total variance for the male and female models, respectively.

## Discussion

This study, to the best of our knowledge, is among the first nationwide large–scale survey at national and regional levels to empirically examine perspectives from a diverse sample of both young men’s and women’s fertility intentions after the government announced a three-child policy shift to curb the dramatic decline in births. The study received responses from participants from all regions of mainland China and representing diverse demographic groups. Association between fertility intentions with childbearing– and childbirth-related anxiety and parenthood–related anxiety were established.

The profound shift in China’s population control programme, allowing couples to have three children, did not receive strong support from young people in this study. Our finding is in concordance with news reports on public reactions to the announcement of the new policy allowing couples to have three children [15–17]. An alarming finding of this study is that low fertility intentions were reported by our study participants across all the regions in China. A recently published study conducted among the public of child-bearing-age between 31 to 40 years in China reported 12.2% had third birth intention [18]. Further, the 2017 National Fertility Survey reported only 2.2% do not plan to have any children [6]. Our study participants were university students of relatively younger age, hence perhaps explaining the relatively lower fertility intentions and higher proportion reported do not wish to have any children in the future. Uncertainty about economic conditions brought on by COVID-19, mental health burden, fear of infection, and high mortality rate from COVID-19 are negatively impacting future birth rates [19]. It is also of important note that the COVID-19 pandemic has caused an upsurge of economic and future uncertainty in China [20], and this may also impact participants’ decision to have children. As evident in China, new births plunged to their lowest in almost six decades in the year 2020 amid the uncertainties of the COVID-19 pandemic [21].

This study also specifically highlights the gendered nature of individuals’ fertility intentions. Male participants reported relatively higher fertility intentions compared to females. The general low fertility intentions among young people indicate that without wide-ranging supportive measures aimed at encouraging births, the country may face a continuing decline in fertility rates in the coming decades. Fertility intentions among young people are the most important drivers that affect fertility behaviour and future fertility trends. Without concerted and courageous action targeting young people, the country is likely moving towards a similar trend as in many European countries [22] and other Asian countries such as Japan, South Korea and Singapore [23] where fertility continued to fall or remained low for prolonged periods.

Little is known about young people’s knowledge and awareness of RMNCH support and/or services in China. The low level of knowledge of RMNCH support and/or services found in this study is fuelling concerns, hence underscoring the importance of young people not being an overlooked population group in future awareness-raising activities in the event the country implements new or improved policies formulated to stimulate birth rates. More importantly, the study also revealed that young people have very limited knowledge even in the most basic RMNCH support and/or services, including pregnancy and childbirth-related services as well as paternal and maternal leave policies. Inadequate knowledge and misconceptions create apprehension about childbearing. A study reported that providing fertility-related information contributes to greater reproductive knowledge and leads to higher childbearing intentions [24]. Therefore, providing young people with information about RMNCH support and/or services may likely foster positive attitudes towards marriage and nurture their beliefs in positive aspects of family building and having children. We also viewed that having greater knowledge of RMNCH support and/or services implemented by the government as supportive measures to promote births holds the potential to facilitate more informed reproductive decision–making. Additionally, our findings also suggest that bridging knowledge gaps is important in consideration of the critical knowledge deficit among young people from lower socio-economic backgrounds.

The young women in this study expressed a high level of childbearing- and childbirth-related anxiety. Specifically, this study found the birthing process as the most important fear underpinning young women’s childbearing- and childbirth-related anxiety. Thus, early intervention is important to ameliorate its negative effects on young women’s future fertility behaviour. Likewise reported in many studies globally, fear or anxiety about the process of labour and delivery was common and was found to deter women’s birth decisions [25, 26]. Fear of childbirth may result in women feeling doubtful of their ability to bear and give birth to a child [27]. Additionally, anxiety in labour also has a significant effect on women’s well–being during pregnancy and their birth outcomes [28]. Other detrimental effects such as postpartum depression, symptoms of post-traumatic stress disorder, and delayed bonding with infants were also reported [29]. In China, although fear of childbirth has never been investigated in women, a study found childbirth fear among pregnant Chinese women, particularly of younger maternal age, was prominent [30]. Various psychological, educational and alternative interventions have been used to reduce fear of childbirth in pregnant women [31], which can be adapted for use in interventions for young women in China.

In many developed countries facing declining fertility, policies to support families to lessen childcare concerns, facilitating work-family reconciliation, and various financial incentives such as childcare subsidies have been found to encourage the decision to have children [32]. Evidence from this study also points towards the benefit of these policies being implemented or improved in China. In the current study, high parenthood anxiety was also found in both men and women. In particular, a high proportion reported the balance between work and childcare as their utmost concern in parenthood, and this was followed by the costs of parenthood. Hence, greater emphasis needs to be placed on overcoming the identified concern, specifically providing a solution to the issues of reconciling work and family life and promoting a work-life balance.

The hypothesis regarding the influence of knowledge about RMNCH support and/or services, childbearing– and childbirth-related anxiety, parenthood–related anxiety on participants’ fertility intentions were confirmed in this study. PLS-SEM showed that lower knowledge of RMNCH support and/or services, and a high level of childbearing– and childbirth-related anxiety and parenthood–related anxiety would result in lower fertility intentions. The models also showed that both childbearing– and childbirth-related anxiety and parenthood anxiety have strong predictive power in fertility intentions, while knowledge has weaker predictive power. In females, both childbearing– and childbirth-related anxiety and parenthood–related anxiety have equal path coefficient values implying equal importance in predicting fertility intentions. Findings imply the need to focus on reducing these anxieties in young people. Another point that should be considered is the significant association between age and fertility intentions in the PLS-SEM path analyses. The significantly higher fertility intention among older age young people implies the need for early intervention.

Our study suggests that improvement of birth policies and provision of support measures to relieve couples’ financial and mental burden of raising children from infancy through to college graduation may enhance higher fertility behaviours of the population. However, other factors known to influence couples’ fertility intentions, such as parental opinion about childbearing, intergenerational effects and social norms [33–35] are beyond the scope of this study. These factors warrant further investigations into their influences on fertility intention. Additional perspectives about these factors must be identified in future investigations.

### Limitations

Our current study sheds light on young people’s fertility intentions and their associated concerns. Nonetheless, the study has some limitations that should be acknowledged and considered. First, the use of an online survey may have resulted in sampling bias, so the results may not be generalizable to the wider community of young people, as reflected in the lack of representation from some locations. In addition, online surveys may not reach individuals who are not in higher education institutions, reside outside internet coverage areas, or lack access to a cell phone. Therefore, young people in rural and underserved communities may be underrepresented in this study. In particular, the response rate from the Southwest, Northeast and Northwest regions were relatively low. Nonetheless, the number of samples in all regions exceeds the calculated optimal sample size. It is noteworthy to mention that this study did not query other important information that may potentially influence fertility intentions such as whether or not participants are in a dating relationship. Second, the issue of self–reporting bias may represent a potential problem in the validity of the assessment. Moreover, it should be noted that although fertility intentions have been found to predict actual birth rates [36], fertility intentions may not coincide with actual fertility behaviour among our study participants. Therefore, the limitations of the current study need to be considered when interpreting the results. Despite the above-mentioned limitations, this study identifies potential fruitful directions for policymakers to improve young people’s understanding of the RMNCH support measures provided by the government. It is also important to note that the Theory of Planned Behavior (TPB), an extensive psychosocial theory used in understanding fertility decisions [37], was not applied in the planning of this study. The significant influence of anxiety towards childbearing and parenthood on fertility intentions in the present study provides potential direction for future research into TPB-based applications in fertility decisions in the new era of the three-child policy in China. Future research is also warranted to explore differences between fertility desires and intentions and to investigate the gap between desired or intended fertility and actual fertility to inform fertility-related policies [38].

## Conclusion

Despite easing birth restrictions to boost the country’s stagnating population growth, the majority of young people have low fertility intentions. This study contributes to providing evidence of the importance of childbearing- and childbirth-related anxiety, parenthood–related anxiety, and knowledge of the RMNCH support and/or services influencing people’s fertility intention. The need for awareness-raising and dissemination of information to increase understanding of RMNCH support and/or services among young people has been demonstrated. The findings also highlighted the importance of early psychological and social support in the childbearing process in women to help them mentally and emotionally prepare for the transition to parenthood. Enforcement of family-friendly policies to cultivate positive parenting and work-life balance is crucial. Financial support to cope with the rising cost of parenthood and reduce economic pressure is imperative. Priorities should be given to the determinants of fertility intentions found in this study in facilitating the achievement of the targeted fertility rate in the country.

## Supplementary Information

**Additional file 1: Supplementary file 1.** Questionnaire.

**Additional file 2: Supplementary file 2.** Factors associated with knowledge about reproductive, maternal, newborn, and child health (RMNCH) support or services.

## Data Availability

The datasets used and/or analyzed during the current study are available from the corresponding author on reasonable request. The datasets generated and/or analysed during the current study are available in the Kaggle repository, [http://www.kaggle.com/dataset/fbf7b6d576d9b04bc98182a88b29661c0c90fa387041121b8fab97940f9db667].

## Abbreviations

RMNCH: Reproductive, maternal, newborn, and child health
CPC: Communist Party of China
PLS-SEM: Partial least squares structural equation modelling
HTMT: Heterotrait-monotrait
SD: Standard deviation
IQR: Interquartile range
