# Supplementary material for: Fertility intentions among young people in the era of China’s three–child policy: a national survey of university students
Source: BMC Pregnancy Childbirth. 2022 Aug 12;22:637. doi: 10.1186/s12884-022-04873-y (PMC9372952; doi:10.1186/s12884-022-04873-y)
Supplement: Supplementary file 1 — Additional file 1: Supplementary file 1. Questionnaire. [file 12884_2022_4873_MOESM1_ESM.docx]

Supplementary File 1 Questionnaire

**A national survey of students’ views about fertility intention**

Section A

**General information**

| **Demographics** | | |
| --- | --- | --- |
| A1 | Sex | [ ] Male  [ ] Female |
| A2 | Age | ___ years old |
| A3 | What is your current educational status？ | [ ] 1^st^grade  [ ] 2^nd^ grade  [ ] 3^rd^ grade  [ ] 4^th^ grade  [ ] 5^th^ grade  [ ] Postgraduate |
| A4 | Types of your University? | [ ] Undergraduate College  [ ] Vocational College |
| A5 | What is your mother’s highest educational level? | [ ] No formal education  [ ] Primary school  [ ] Junior middle school  [ ] Secondary specialized school  [ ] High school  [ ] Junior college  [ ] Bachelor degree  [ ] Master  [ ] Doctoral |
| A6 | What is your father’s highest educational level? | [ ] No formal education  [ ] Primary school  [ ] Junior middle school  [ ] Secondary specialized school  [ ] High school  [ ] Junior college  [ ] Bachelor degree  [ ] Master  [ ] Doctoral |
| A7 | Your family average monthly household income in CNY | [ ] <4000  [ ] 4000-9999  [ ] 10000-14999  [ ] 15000-19999  [ ] ≥20000 |
| A8 | Which region are you from？ | [ ] Northern region (Beijing, Tianjian, Hebei, Shanxi, Inner Mongolia)  [ ] Northeast China (Liaoning, Jilin, Heilongjiang)  [ ] East region (Shanghai, Jiangsu, Zhejiang, Anhui, Fujian, Jiangxi, Shandong, );  [ ] Southern Central China (Hunan, Guangdong, Guangxi, Hainan, Henan, Hubei);  [ ] Southwest China (Chongqing, Sichuan, Guizhou, Yunnan, Tibet);  [ ] Northwest China (Shannxi, Gansu, Qinghai, Ningxia, Xinjiang) |

Section B

**Fertility intention**

|  | | |
| --- | --- | --- |
| B1 | If you are starting your family in the future, how many children would you want altogether? | [ ] I do not wish to start a family in the future  [ ] I do not wish to have any children  [ ] 1  [ ] 2  [ ] 3  [ ] >3 |

Section C

**Childbearing- and childbirth-related anxiety（FEMALE ONLY）**

| C1 | On the scale of 0 to 10, overall, how would rate your anxiety about childbearing and childbirth? [higher the value, the higher anxiety] | 0, 1, 2, 3, 4, 5, 6, 7, 8, 9, 10 |
| --- | --- | --- |
| C2 | Which of the following is the most fearful regarding childbearing and childbirth? | [ ] Childbearing/pregnancy process  [ ] Delivery process  [ ] Personal health risk  [ ] Infant’s health (fetal anomalies/birth defects)  [ ] Cost/ associated expenses |

Section D

**Parenthood-related anxiety**

| D1 | On the scale of 0 to 10, overall, how would rate your anxiety related to parenthood? [higher the value, the higher anxiety] | 0, 1, 2, 3, 4, 5, 6, 7, 8, 9, 10 |
| --- | --- | --- |
| D2 | Which of the following is the most fearful regarding parenthood? | [ ] Balancing work and childcare  [ ] Loss of freedom  [ ] Loss of self-identity  [ ] Interfere relationship with spouse  [ ] Cost/associated expenses |

Section E

**Knowledge about reproductive, maternal, newborn, and child health (RMNCH) support and/or services**

| **Please rate your level of knowledge of the reproductive, maternal, newborn, and child health (RMNCH) support or services in your area** | | |
| --- | --- | --- |
| E1 | Antenatal/prenatal (before birth) care services | [ ] Very knowledgeable  [ ] Knowledgeable  [ ] Aware but don’t know much  [ ] Not aware |
| E2 | Perinatal (time around birth) care services | [ ] Very knowledgeable  [ ] Knowledgeable  [ ] Aware but don’t know much  [ ] Not aware |
| E3 | Postnatal (after birth) care services | [ ] Very knowledgeable  [ ] Knowledgeable  [ ] Aware but don’t know much  [ ] Not aware |
| E4 | Infant/baby (≤3 years old) care services | [ ] Very knowledgeable  [ ] Knowledgeable  [ ] Aware but don’t know much  [ ] Not aware |
| E5 | Childcare /pre-primary school (>3 years old) services | [ ] Very knowledgeable  [ ] Knowledgeable  [ ] Aware but don’t know much  [ ] Not aware |
| E6 | Childbirth services (delivery care equipment and facilities, medical specialist in hospital) | [ ] Very knowledgeable  [ ] Knowledgeable  [ ] Aware but don’t know much  [ ] Not aware |
| E7 | Family planning services (contraceptives provision, counseling on gaps of childbirth) | [ ] Very knowledgeable  [ ] Knowledgeable  [ ] Aware but don’t know much  [ ] Not aware |
| E8 | Fertility services or treatment | [ ] Very knowledgeable  [ ] Knowledgeable  [ ] Aware but don’t know much  [ ] Not aware |
| E9 | Maternity leave entitlement | [ ] Very knowledgeable  [ ] Knowledgeable  [ ] Aware but don’t know much  [ ] Not aware |
| ED10 | Paternity leave entitlement | [ ] Very knowledgeable  [ ] Knowledgeable  [ ] Aware but don’t know much  [ ] Not aware |
| E11 | Breastfeeding leave for working mother | [ ] Very knowledgeable  [ ] Knowledgeable  [ ] Aware but don’t know much  [ ] Not aware |
| E12 | Maternity medical insurance system | [ ] Very knowledgeable  [ ] Knowledgeable  [ ] Aware but don’t know much  [ ] Not aware |
| E13 | Female employees' reproductive rights and interests | [ ] Very knowledgeable  [ ] Knowledgeable  [ ] Aware but don’t know much  [ ] Not aware |

**THANK YOU**
