# Supplementary material for: Fertility intentions among young people in the era of China’s three–child policy: a national survey of university students
Source: BMC Pregnancy Childbirth. 2022 Aug 12;22:637. doi: 10.1186/s12884-022-04873-y (PMC9372952; doi:10.1186/s12884-022-04873-y)
Supplement: Supplementary file 2 — Additional file 2: Supplementary file 2. Factors associated with knowledge about reproductive, maternal, newborn, and child health (RMNCH) support or services. [file 12884_2022_4873_MOESM2_ESM.docx]

Supplementary File 2 Factors associated with knowledge about reproductive, maternal, newborn, and child health (RMNCH) support or services

|  | OR  Knowledge score 8–39 *vs* 0–7 | 95% CI | *P* |
| --- | --- | --- | --- |
| **Socio demographic characteristics** |  |  |  |
| Age group (years) |  |  |  |
| 18–20 | 1.01 | 0.85–1.18 | 0.952 |
| 21–23 | 1.23 | 1.04–1.45 | 0.016 |
| 24–28 | 1 |  |  |
| Gender |  |  |  |
| Male | 1.59 | 1.44-1.75 | p<0.001 |
| Female | 1 |  |  |
| Grade |  |  |  |
| Grade 1 | 1.12 | 0.93–1.36 | 0.243 |
| Grade 2 | 1.27 | 1.09–1.48 | 0.002 |
| Grade 3 | 1.37 | 1.17–1.61 | p<0.001 |
| Grade 4/5 | 1.37 | 1.16–1.62 | p<0.001 |
| Postgraduate | 1 |  |  |
| Maternal highest education level |  |  |  |
| Primary school | 0.49 | 0.42–0.56 | p<0.001 |
| Junior middle school | 0.63 | 0.55–0.73 | p<0.001 |
| Secondary school/ high school | 0.88 | 0.75–1.03 | 0.106 |
| College/ university | 1 |  |  |
| Paternal highest education |  |  |  |
| Primary school | 0.60 | 0.51–0.69 | p<0.001 |
| Junior middle school | 0.68 | 0.60–0.77 | p<0.001 |
| Secondary school/ high school | 1.00 | 0.87–1.16 | 0.969 |
| College/ university | 1 |  |  |
| Monthly household income (CNY¥) |  |  |  |
| <4000 | 0.52 | 0.44–0.62 | p<0.001 |
| 4000–9999 | 0.68 | 0.59–0.79 | p<0.001 |
| 10000–14999 | 0.79 | 0.67–0.93 | 0.05 |
| 15000 and above | 1 |  |  |
| Region |  |  |  |
| Northern | 0.93 | 0.76-1.14 | 0.482 |
| Northeastern | 1.03 | 0.84-1.27 | 0.763 |
| Eastern | 0.62 | 0.53-0.73 | p<0.001 |
| Southern Central | 0.66 | 0.54-0.81 | p<0.001 |
| Southwestern | 0.91 | 0.75-1.12 | 0.375 |
| Northwestern | 1 |  |  |

Note: The normality distribution of theknowledge score using the Kolmogorov–Smirnov test was found that the data were not normally distributed (*p <*0.05). Dichotomization of total knowledge scores were done using median split to form high (score 8–39) and low score (score 0–7) groups.
